# Dual-vector prodrug activator gene therapy using retroviral replicating vectors

**DOI:** 10.1038/s41417-018-0051-0

**Published:** 2018-10-22

**Authors:** Shuji Kubo, Misato Takagi-Kimura, Masatoshi Tagawa, Noriyuki Kasahara

**Affiliations:** 1 Unit of Molecular and Genetic Therapeutics, Laboratory of Medical Innovation, Institute for Advanced Medical Sciences, Hyogo College of Medicine, Nishinomiya, Japan; 2 Division of Pathology and Cell Therapy, Chiba Cancer Center Research Institute, Chiba, Japan; 3 Department of Cell Biology and Pathology, University of Miami, Miami, USA

**Keywords:** Genetic vectors, Cancer therapy

## Abstract

Retroviral replicating vectors (RRVs) have been shown to achieve efficient tumor transduction and enhanced therapeutic benefits in a variety of cancer models. In the present study, we evaluated a possible combinatorial effect of prodrug activator genes delivered by two different RRVs derived from amphotropic murine leukemia virus (AMLV) and gibbon ape leukemia virus (GALV) on human hepatocellular carcinoma Hep3B cells. Both RRVs showed efficient replicative spread in culture and can overcame superinfection resistance each other. Notably, the replication and spread of each RRV in culture remained unaffected by pretransduction with the counterpart RRV. We further transduced cells with RRVs which individually possessed the prodrug activator genes yeast cytosine deaminase (CD) and herpes simplex virus thymidine kinase (TK) alone or in combination, and evaluated the cytotoxic effects of RRV-mediated gene therapy with CD and TK in the presence of the respective prodrugs, 5-fluorocytosine and ganciclovir. All combinations of the two prodrug activator genes produced synergistic cytocidal effects, but the combined effects of the different genes were significantly greater than those of the same genes when delivered by two different vectors. The present findings indicate the potential utility of dual-vector gene therapy using two different RRVs carrying different prodrug activator genes.

## Introduction

Advances in gene transfer technologies have opened the way toward cancer treatment by gene therapy. However, a number of clinical trials on cancer gene therapy have been performed over the past few decades without satisfactory results [1, 2]. The major drawback has been the low efficiency for delivery of therapeutic genes, such as prodrug activator genes, using conventional replication-defective viral vectors [3–5]. To address this point, replication-competent vectors are currently attracting much attention for oncolytic virotherapy because of their expected higher efficiency, given that each successfully transduced cancer cell itself becomes a virus-producing cell, thereby sustaining further transduction events after the initial administration [6, 7].

We previously developed retroviral replicating vectors (RRVs) that can achieve tumor-selective transduction, because of their inability to infect non-dividing cells and the defective antiviral defense in most cancer cells [8–15]. In contrast to other viruses used in cancer virotherapy, RRVs are non-cytolytic, but can be engineered to carry prodrug activator genes that mediate synchronized cell killing of infected tumor cells upon prodrug administration. Using RRVs that express yeast cytosine deaminase (CD), which converts the prodrug 5-fluorocytosine (5FC) to the toxic metabolite 5-fluorouracil, we showed highly efficient killing of a wide variety of cancer cells in vitro and in vivo following 5FC administration [10–12, 16–19]. Based on these promising preclinical results, a phase 1 clinical trial for RRV-mediated prodrug activator gene therapy in patients with recurrent glioblastoma was recently conducted in the United States and Europe, resulting in long-term survival and systemic antitumor immunity mediated by memory T cells [20]. RRVs are currently under evaluation in an international phase III trial for recurrent high-grade glioma.

To date, we have developed two different RRVs derived from amphotropic murine leukemia virus (AMLV) and gibbon ape leukemia virus (GALV) [12, 13]. Although AMLV and GALV are both gammaretroviruses, they have divergent host ranges and are in different interference classes because they employ different receptors to infect target cells [21–23]. Specifically, AMLV uses cellular receptor PiT2 (SLC20A2), while GALV uses PiT1 (SLC20A1). These two receptors are mammalian type III inorganic phosphate transporters that are present in all phyla and function as ubiquitously expressed facilitators of phosphate uptake [21–23]. We previously reported that most cancer cells express both PiT1 and PiT2, while some cancer cell lines express them differentially [12]. However, the mechanisms and physiological effects of this differential expression of phosphate transporters remain to be elucidated. In cells with low receptor expression, RRVs requiring the receptor for viral entry showed limited replicative spread, and the efficiency of cell death after prodrug administration was correlated with the RRV replication kinetics. Therefore, the use of multiple RRVs requiring different receptors may be practically beneficial in RRV-mediated prodrug activator gene therapy against a variety of solid tumors.

Prodrug activator genes, also known as suicide genes, transduced into target cancer cells convert inactive prodrugs into cytotoxic metabolites (chemotherapeutic agents) for the cancer cells [24, 25]. RRV-mediated prodrug activator gene therapy would be the ultimate “intracellular” chemotherapy, and fairly well restricted to tumor cells, resulting in fewer adverse side effects. Nevertheless, cancer cells eventually become resistant to any single chemotherapeutic agent. Thus, in an ideal clinical setting, combination regimens are essential for long-term success to reduce the risk of resistance and presumably enhance the antitumor effect by additive or synergetic effects. For this purpose, we created RRVs encoding different prodrug activator genes, comprising CD [10, 19] and herpes simplex virus thymidine kinase (TK). However, when RRV-mediated prodrug activator gene therapy against a solid tumor fails to achieve satisfactory results, the same viral strain cannot be used to infect and transduce the tumor cells. This resistance to serial infection is caused by receptor interference, and results in superinfection resistance [26].

Our study objective was to provide proof of concept for dual-vector prodrug activator gene therapy using AMLV/GALV-RRVs for solid cancers. For this, we evaluated whether two different AMLV/GALV-RRVs can overcome superinfection resistance without interfering with each other’s replication and spread in culture. In addition, we investigated whether the combination of AMLV/GALV-RRVs can exert an enhanced in vitro cytotoxic effect against tumor cells.

## Materials and methods

### Cell lines

Human hepatocellular carcinoma Hep3B cells and 293T cell lines (ATCC, Manassas, VA, USA) were cultured in Dulbecco’s modified Eagle’s medium (Nacalai Tesque, Kyoto, Japan) supplemented with 10% fetal bovine serum at 37 °C under 5% CO_2_.

### Viral vector plasmid and virus production

**Fig. 1:**
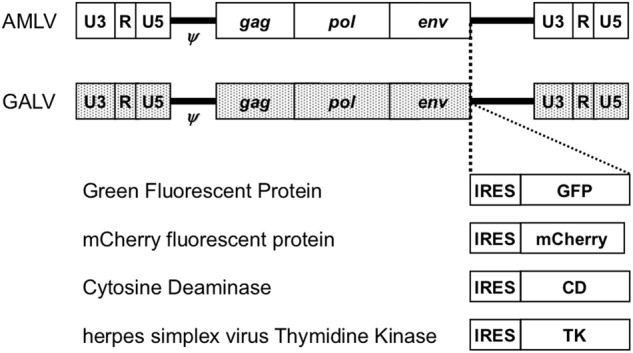
Schematic structures of the RRVs. The vectors contain a full-length replication-competent AMLV or GALV provirus, in which an IRES-transgene cassette has been inserted between the *env* gene and the 3′-untranslated region. *ψ*: packaging signal; *gag-pol*: AMLV or GALV structural genes; IRES: internal ribosome entry site

The RRV plasmids, pAMLV-GFP, pGALV-GFP, pAMLV-CD, and pGALV-CD, were described previously [12]. Each vector contained a full-length replication-competent amphotropic AMLV or GALV provirus with an additional internal ribosome entry site (IRES)-GFP or IRES-CD cassette (Fig. 1). pAMLV-mCherry, pGALV-mCherry, pAMLV-TK, and pGALV-TK were generated by replacement of the IRES-GFP sequence in pAMLV-GFP or pGALV-GFP with an IRES-mCherry or IRES-TK cassette (Fig. 1). For virus production, 293T cells were transiently transfected with a vector plasmid using Lipofectamine 2000 (Life Technologies Japan, Tokyo, Japan), replenished with serum-free medium, and cultured for 48 h. The supernatant medium was then harvested, filtered, and stored at −80 °C [10, 12]. The titers of the vectors were determined by fluorescent protein expression using a FACSCalibur flow cytometer (Becton Dickinson Japan, Tokyo, Japan) and expressed as transducing units (TU) per milliliter.

### Replication kinetics of RRVs in vitro

For analysis of replication kinetics in vitro after single RRV infection, Hep3B cells at 20% confluency were infected with AMLV-GFP or GALV-GFP at a multiplicity of infection (MOI) of 0.01. At serial time points, the cells were trypsinized, and one-fourth of the cells were replated. The remaining cells were analyzed for GFP expression by flow cytometry as described above.

For serial infection, cells pretransduced with AMLV-mCherry or GALV-mCherry at an MOI of 0.01 and maintained for 10 days were infected with AMLV-GFP or GALV-GFP at 20% confluency in six-well plates. At serial time points, the cells were analyzed for GFP and mCherry expression by flow cytometry.

### Cytotoxicity assay in vitro

For quantitative analysis of drug cytotoxicity, triplicate wells containing Hep3B cells (1 × 10^4^ cells/well) that had been pretransduced with AMLV-CD and/or GALV-CD at an MOI of 0.01 and maintained for 10 days were cultured in 96-well tissue-culture plates with various concentrations of 5FC (Wako Pure Chemical Industries, Osaka, Japan) and ganciclovir (GCV; Wako Pure Chemical Industries). On day 3, the viable cell numbers in the triplicate cultures were measured using Alamar Blue (Alamar Biosciences Inc., Sacramento, CA, USA) according to the manufacturer’s instructions. Briefly, 40 μL of Alamar Blue was aseptically added to the cultures, and the plates were returned to the incubator for 3 h. Fluorescence was measured by an ARVO X4 multilabel plate reader (PerkinElmer Japan, Tokyo, Japan) with excitation wavelength of 544 nm and emission wavelength of 590 nm. The percentage of viable cells was determined by calculation of viable cell fluorescence in wells containing 5FC and GCV relative to that in wells without 5FC and GCV. Combinatorial effects were assessed by combination index (CI) values using CalcuSyn software (Biosoft, Cambridge, UK) in different fraction-affected points, to show the relative levels of suppressed cell viability. Values of CI < 1, CI = 1, and CI > 1 were considered to indicate synergistic, additive, and antagonistic actions, respectively.

### Statistical analysis

Data are presented as means ± SD. The statistical significance of differences in data was evaluated by Student’s *t*-test and ANOVA. Values of *P* < 0.05 were considered significant in all analyses.

## Results

### RRVs replicate efficiently in human hepatocellular carcinoma Hep3B cells

**Fig. 2:**
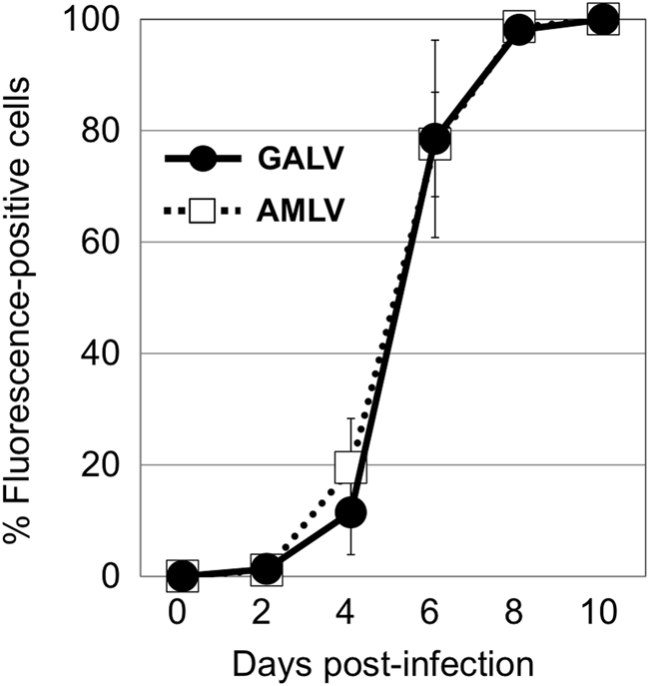
Replication kinetics of RRVs in Hep3B cells. Hep3B cells were inoculated with AMLV-GFP or GALV-GFP at a multiplicity of infection of 0.01. On passage days, the cells were analyzed for GFP expression by flow cytometry. Data shown are means ± SD from triplicate experiments

First, we screened the expression levels of the cellular receptors for GALV (PiT1) and AMLV (PiT2) in human cell lines by qPCR (data not shown), and found that the mRNA levels of both receptors were high in most cancer cell lines, including Hep3B cells (>100-fold compared with fibroblasts). To evaluate the replication kinetics in Hep3B cells, we used RRVs expressing the GFP gene (AMLV-GFP and GALV-GFP; Fig. 1). GFP expression was monitored by flow cytometry at serial time points after inoculation of cells with AMLV-GFP or GALV-GFP at an MOI of 0.01 (Fig. 2). Both RRVs efficiently replicated in Hep3B cells and spread in culture with similar kinetics (Fig. 2). Thus, Hep3B cells were selected for further experiments.

### Superinfection resistance and its resolution by different RRVs that use different cellular receptors

**Fig. 3:**
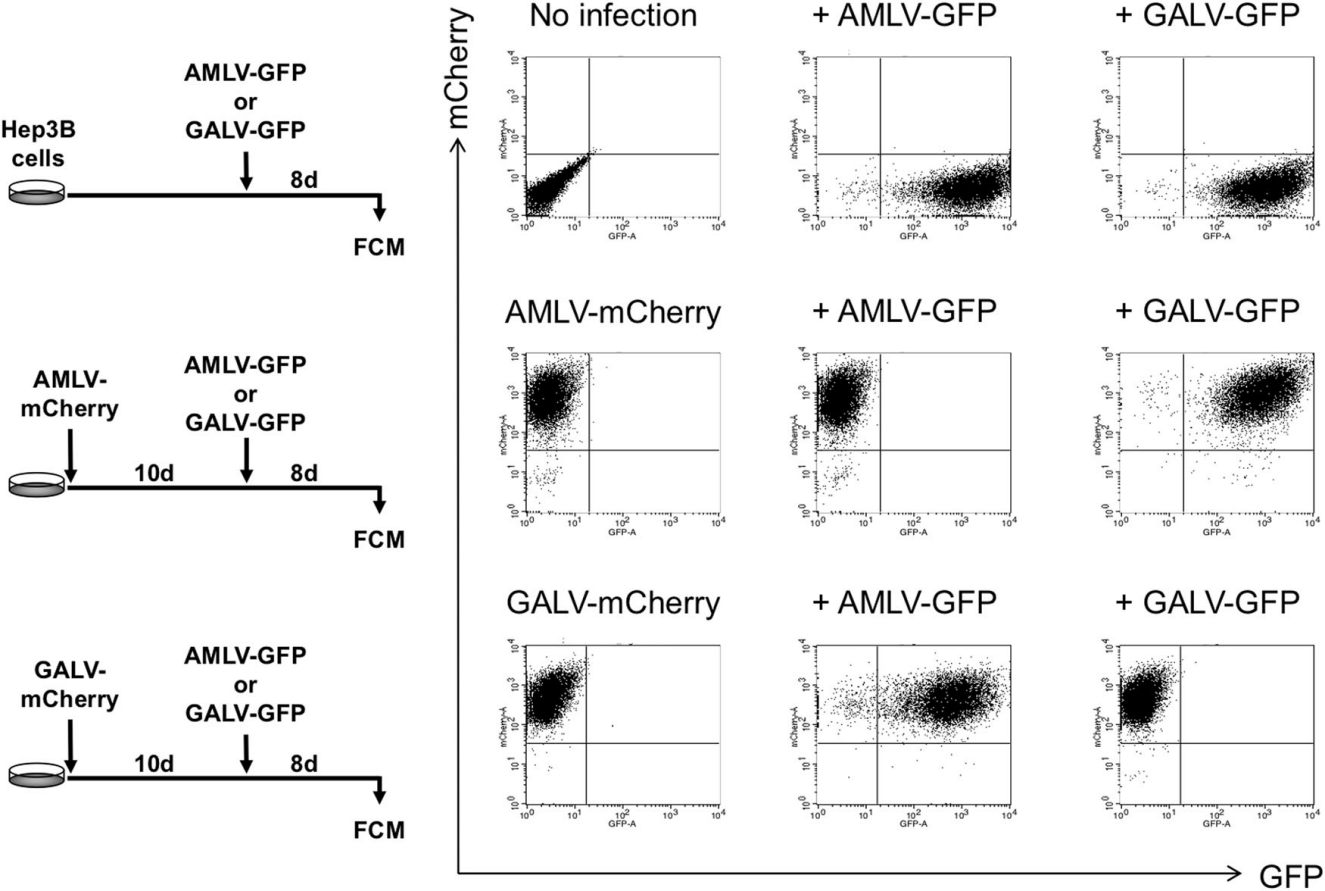
Transduction of Hep3B cells with RRVs. Following single infection, both AMLV-GFP and GALV-GFP were able to infect Hep3B cells and spread in culture (top). When Hep3B cells were pretransduced with AMLV-mCherry, GALV-GFP, but not AMLV-GFP, was able to infect the cells and spread in culture (middle). Conversely, when Hep3B cells were pretransduced with GALV-mCherry, AMLV-GFP, but not GALV-GFP, was able to infect the cells and spread in culture (bottom). FCM flow cytometry

Next, we confirmed that the RRVs could serially infect Hep3B cells and spread in culture. Following single infection, AMLV-GFP and GALV-GFP successfully infected Hep3B cells and spread in culture (Fig. 3, upper). When Hep3B cells were pretransduced with AMLV-mCherry, GALV-GFP, but not AMLV-GFP, was able to infect the cells and spread in culture (Fig. 3, middle). Conversely, when Hep3B cells were pretransduced with GALV-mCherry, AMLV-GFP, but not GALV-GFP, was able to infect the cells and spread in culture (Fig. 3, bottom). This mutually exclusive infection pattern arose through receptor interference, resulting in superinfection resistance when the same viral strain was used. These data indicate the potential utility of dual-vector gene therapy with two different RRVs that use different cellular receptors.

### AMLV and GALV do not interfere with each other’s replication and spread in culture

**Fig. 4:**
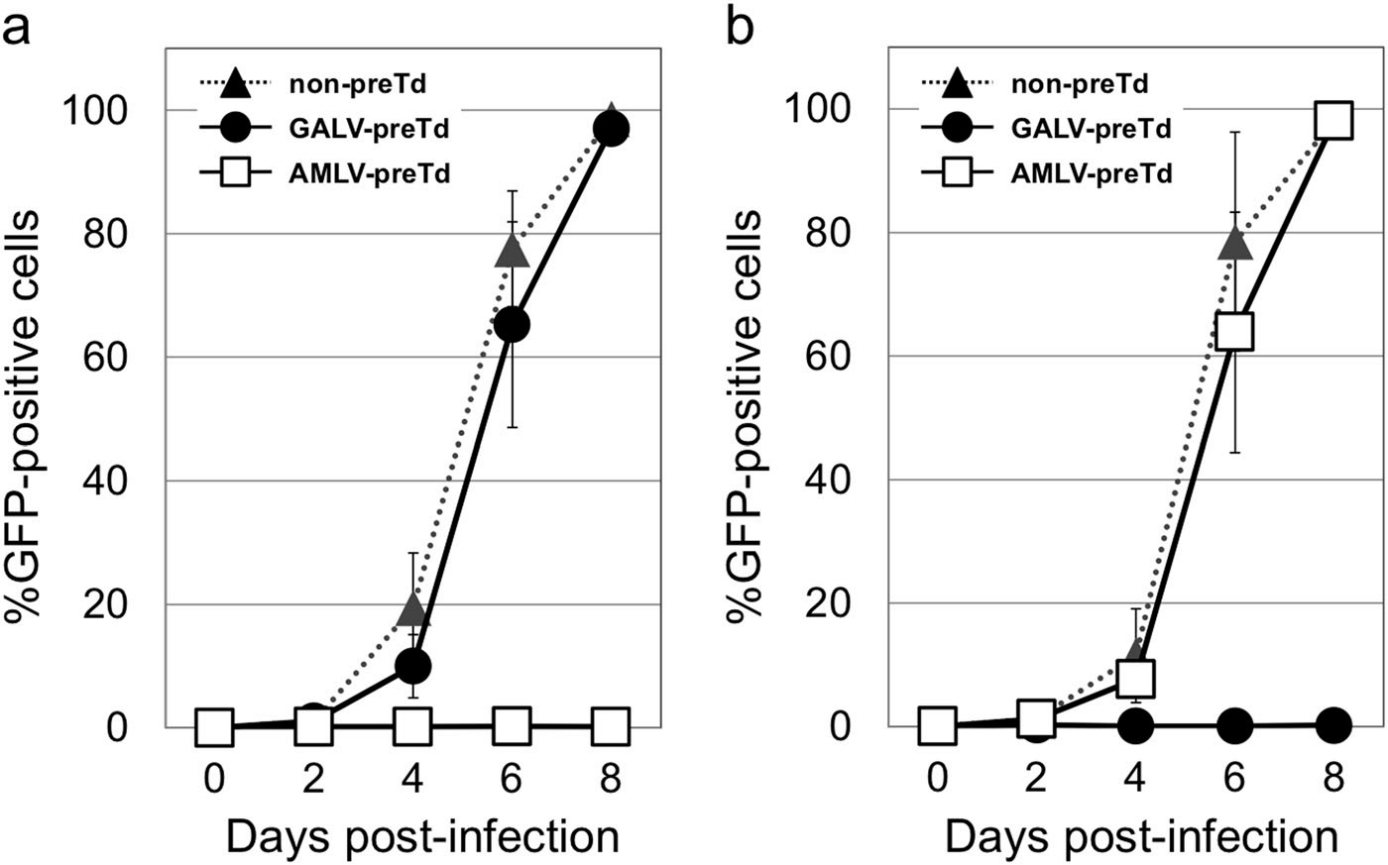
Replication kinetics of RRVs in Hep3B cells with and without pretransduction. **a** Replication and spread of AMLV-GFP in culture were comparable between cells with and without GALV-mCherry pretransduction. Data shown are means ± SD from triplicate experiments. **b** Replication and spread of GALV-GFP in culture were comparable between cells with and without AMLV-mCherry pretransduction. Data shown are means ± SD from triplicate experiments. preTd pretransduced

We further confirmed that the two RRVs did not interfere with each other’s replication and spread in culture. As shown in Fig. 4a, the replication and spread of AMLV-GFP in culture were comparable between cells with and without GALV-mCherry pretransduction. Similarly, the replication and spread of GALV-GFP in culture were comparable between cells with or without AMLV-mCherry pretransduction (Fig. 4b). Similar results were obtained using various other human cancer cell lines (i.e., HepG2 hepatocellular carcinoma, MNNG-HOS osteosarcoma, and MSTO-211H mesothelioma cells) (data not shown). These data showed that the replication and spread of each RRV in culture were not affected by pretransduction with the counterpart RRV.

### Combination effect of dual-RRV prodrug activator gene therapy

**Fig. 5:**
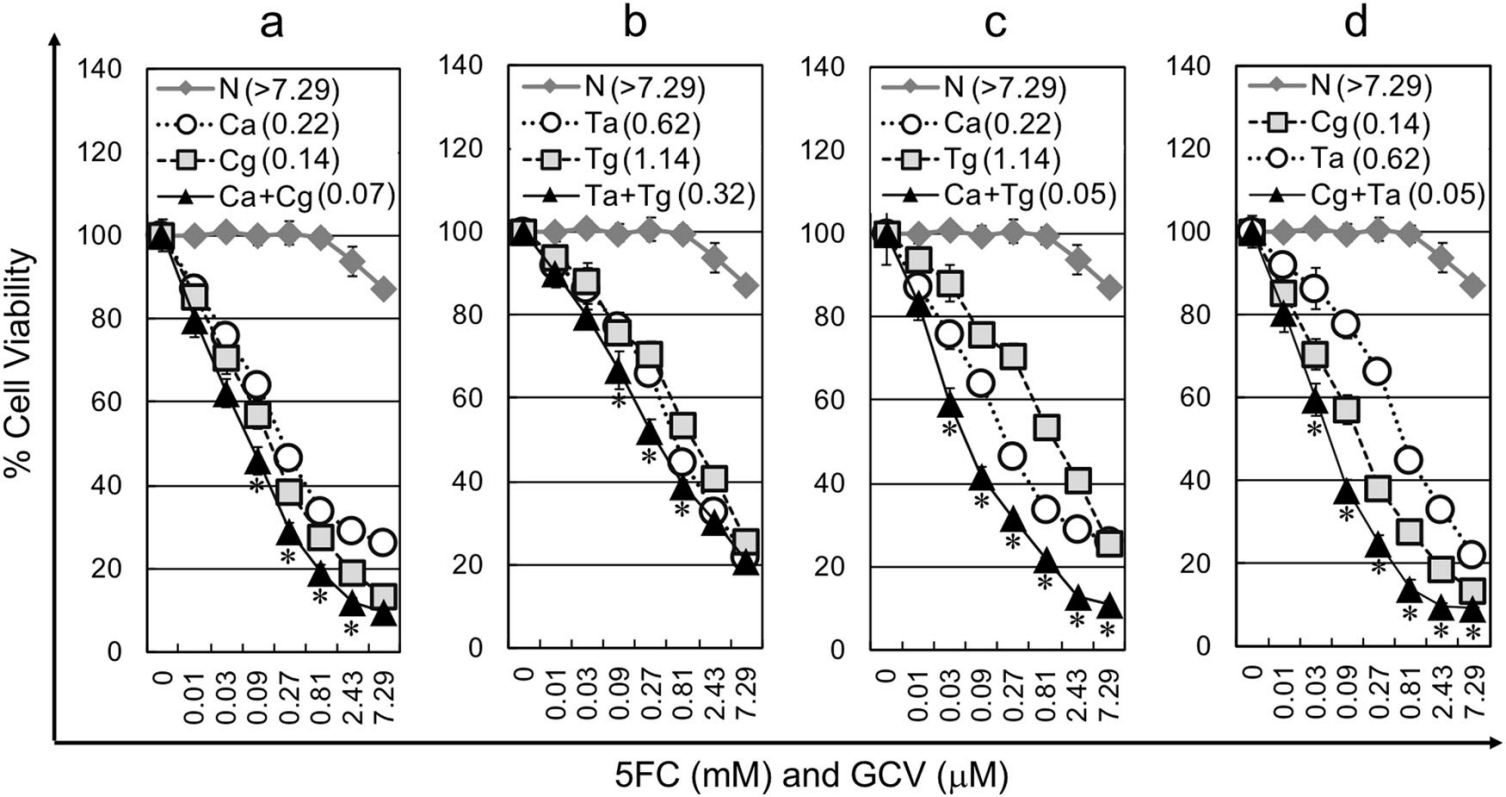
Cytotoxic effect of single-RRV or dual-RRV prodrug activator gene therapy after RRV infection in vitro. The cell viabilities of RRV-pretransduced Hep3B cells on day 3 after exposure to 5FC (mM) and GCV (μM) were evaluated by the Alamar Blue assay. Data shown are means ± SD from triplicate experiments. The combination groups for infection were CD and CD (**a**), TK and TK (**b**), and CD and TK (**c**, **d**). N: No infection; Ca: AMLV-CD; Cg: GALV-CD; Ca + Cg: AMLV-CD + GALV-CD; Ta: AMLV-TK; Tg: GALV-TK; Ta + Tg: AMLV-TK + GALV-TK; Ca + Tg: AMLV-CD + GALV-TK; Cg + Ta: GALV-CD + AMLV-TK. IC_50_ values were shown in parentheses. *P < 0.05 (combination vs. any single)

**Fig. 6:**
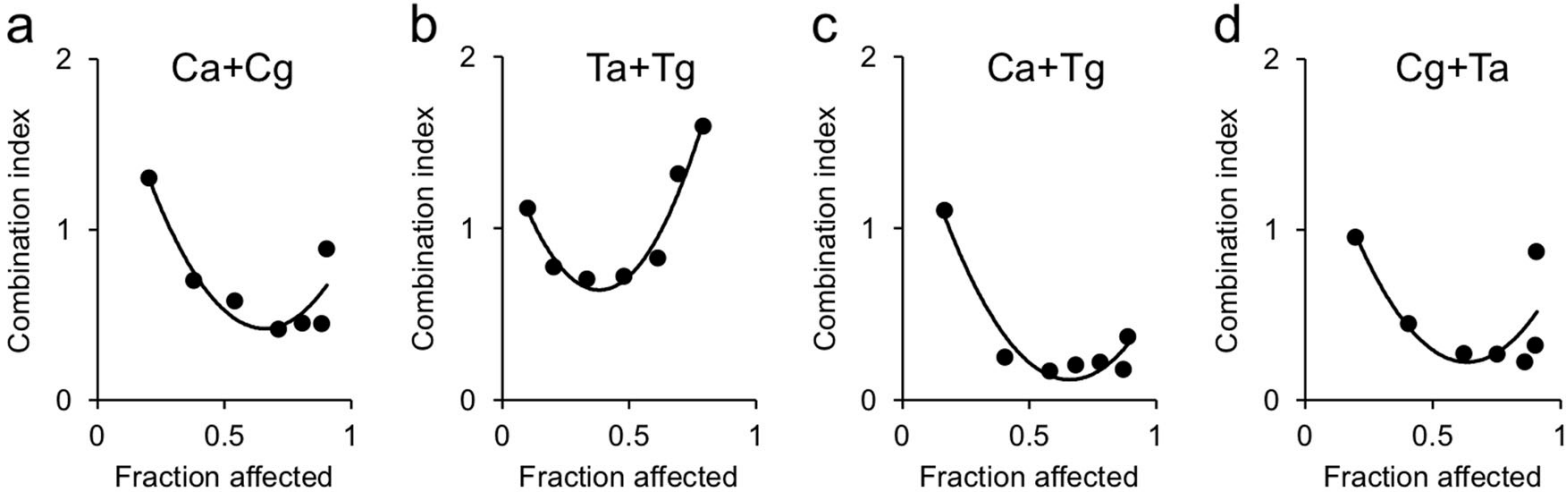
Combinatorial effects of dual-RRV prodrug activator gene therapy in vitro. Combination index values based on the cell viabilities in the experiments shown in Fig. 5 were calculated at different fraction-affected points with CalcuSyn software. The combination groups for infection were CD and CD (**a**), TK and TK (**b**), and CD and TK (**c**, **d**). Ca + Cg: AMLV-CD + GALV-CD; Ta + Tg: AMLV-TK + GALV-TK; Ca + Tg: AMLV-CD + GALV-TK; Cg + Ta: GALV-CD + AMLV-TK

To investigate the effect of combined prodrug-dependent cell killing versus multiple vector copy transduction in vitro, Hep3B cells were transduced with AMLV-CD, GALV-CD, AMLV-TK, and GALV-TK alone or in combination. The resultant cells were evaluated for the cytotoxic effect of RRV-mediated prodrug activator gene therapy with CD and TK in the presence of the respective prodrugs, 5FC and GCV. The combined in vitro cytocidal effects for the same prodrug activator genes were significantly greater than those for a single prodrug activator gene when delivered by a single RRV in most of the dose points (*P* < 0.05 in Ca + Cg between 0.09 and 2.43 in Fig. 5a, in Ta + Tg between 0.03 and 0.81 in Fig. 5b, in Ca + Tg between 0.03 and 7.29 in Fig. 5c and in Cg + Ta between 0.03 and 7.29 in Fig. 5d). Notably, the combined cytocidal effects for different prodrug activator genes were greater than those for the same prodrug activator genes when delivered by two different vectors (Fig. 5c, d). To assess the combinatorial effects of the prodrug activator genes, we calculated CI values at several fraction-affected points corresponding to the percentages of viable cells evaluated by the Alamar Blue assay (Fig. 6). The CI values for all combinations of prodrug activator genes were less than 1 in the majority of the fraction-affected points tested, indicating synergistic effects. Furthermore, the CI values for the combinations “Ca + Tg” (Fig. 6c) and “Cg + Ta” (Fig. 6d) were much lower than those for the combinations “Ca + Cg” (Fig. 6a) and “Ta + Tg” (Fig. 6b). *P* values by ANOVA using CI values of the fraction-affected points between 0.2 and 0.9 were *P* < 0.05 in Ca + Cg vs. Ca + Tg, Ca + Cg vs. Cg + Ta, Ta + Tg vs. Ca + Tg and Ta + Tg vs. Cg + Ta, demonstrating that the combinatorial effects for different prodrug activator genes were significantly greater than those for the same prodrug activator genes when delivered by two different vectors.

The above data indicate the potential utility of dual-vector prodrug activator gene therapy using two different RRVs carrying different prodrug activator genes.

## Discussion

In the present study, we have shown that two different RRVs, AMLV and GALV, utilizing different cellular receptors can overcome superinfection resistance. Furthermore, upon serial infection, the two RRVs did not interfere with each other’s replication and spread in culture. Because AMLV and GALV have low sequence homology [27, 28], the risk of recombination between these RRVs appears low. The present findings suggest that the potential utility of dual-vector combination therapy for solid tumors using combination regimens, which will reduce the risk of resistance and presumably enhance the antitumor effect by additive or synergetic effects.

Our study demonstrated that dual-vector combination therapy resulted in an enhanced in vitro cytocidal effect, even for combinations of the same prodrug activator genes, probably through the increased copy number of the genes in the infected cells. Furthermore, as expected, the combined effects for different prodrug activator genes were much greater than those for the same prodrug activator genes, indicating strong synergistic effects. Dual-prodrug activator gene therapy, TK/GCV and CD/5FC, was reported to show enhanced antitumor activities in vitro and in vivo [29–31]. TK phosphorylates antiviral nucleoside analogs such as GCV, leading to accumulation of its cytotoxic metabolite, GCV triphosphate, with subsequent incorporation into DNA and apoptosis [32, 33]. CD deaminates the prodrug 5FC to form 5-fluorouracil, which is metabolized by cellular enzymes to 5-fluorodeoxyuridylic acid monophosphate that inhibits thymidylate synthase, resulting in depletion of deoxythymidine triphosphate (dTTP) pools and DNA double-strand breaks, and finally leading to cell death [34, 35]. The mechanisms for the synergy between CD/5FC and TK/GCV therapies can be explained by 5FC-mediated reduction of dTTP, which decreases deoxyguanosine triphosphate through allosteric regulation of ribonucleotide reductase, resulting in increased incorporation of GCV triphosphate into DNA to increase cytotoxicity [29–31]. Thus, the dual RRV-mediated CD/5FC and TK/GCV gene therapy could be the ultimate “intracellular” combinatorial chemotherapy to achieve synergistic cell killing, analogous to combination chemotherapy at the intracellular level, and is fairly well restricted to tumors, thus resulting in fewer adverse side effects.

In contrast to other viruses used in cancer virotherapy, RRVs are non-cytolytic and have low immunogenicity, and can thus spread stealthily and efficiently throughout the tumor cells by budding rather than by lysis [8, 15]. RRVs also become permanently integrated into the cancer cell genome, and can stay in the tumor for long time. Tumor cell killing can be induced synchronically by prodrug administration, once the cells have been armed with the prodrug activator genes. These unique features of RRVs allow targeting of cancers for long-term control by optimizing the dose, period, and interval of prodrug administration [18, 20]. Therefore, dual RRV-mediated combinatorial prodrug activator gene therapy provides an alternative to the traditional approach, as a control-based regimen focused on extending patient life and reducing suffering by limiting, rather than eradicating, the growth and spread of cancer, and producing an enhanced antitumor effect while avoiding drug resistance. Additionally, the prodrugs for CD (5FC) and TK (e.g. valganciclovir) are orally available and patients can undergo prodrug administration at outpatient clinics following single injection with RRVs into the tumor. This will be a promising future type of cancer treatment, considering quality of life.

In conclusion, our results demonstrated AMLV and GALV can coinfect and replicate independently in cultured cells, suggesting their ideal combination for dual-vector gene therapy. The dual RRV-mediated CD/5FC and TK/GCV combinatorial gene therapy achieved synergistic cytotoxic efficacy compared with single-vector gene therapy. Thus, coinfection of cancer cells with AMLV and GALV vectors supplied with different prodrug activator genes may be employed for combination intracellular chemotherapy, leading to enhanced cytotoxic effects while avoiding drug resistance.
